# Proton beam therapy for hepatocellular carcinoma with bile duct invasion

**DOI:** 10.1186/s12876-023-02897-y

**Published:** 2023-08-03

**Authors:** Takashi Iizumi, Toshiyuki Okumura, Naoyuki Hasegawa, Kazunori Ishige, Kuniaki Fukuda, Emiko Seo, Hirokazu Makishima, Hikaru Niitsu, Mizuki Takahashi, Yuta Sekino, Hiroaki Takahashi, Daichi Takizawa, Yoshiko Oshiro, Keiichiro Baba, Motohiro Murakami, Takashi Saito, Haruko Numajiri, Masashi Mizumoto, Kei Nakai, Hideyuki Sakurai

**Affiliations:** 1 Department of Radiation Oncology and Proton Medical Research Center, University of Tsukuba, 2-1-1 Amakubo, Tsukuba, 305-8576 Ibaraki Japan; 2 Department of Gastroenterology, Faculty of Medicine, University of Tsukuba, Tsukuba, Japan; 3 Department of Gastroenterology, Kasumigaura Medical Center, Ibaraki, Japan; 4 Department of Radiology, Mayo Clinic, Rochester, USA; 5 Department of Radiation Oncology, Hitachi General Hospital, Tsukuba, Japan; 6 Department of Radiation Oncology, Tsukuba Medical Center Hospital, Tsukuba, Japan

**Keywords:** Bile duct invasion, Bile duct tumor thrombosis, Hepatocellular carcinoma, Proton beam therapy, Radiation therapy

## Abstract

**Aim:**

Hepatocellular carcinoma (HCC) with bile duct invasion (BDI) (BDIHCC) has a poor prognosis. Moreover, due to the paucity of reports, there is no consensus regarding optimal management of this clinical condition yet. The aim of this study was to clarify the efficacy and safety of proton beam therapy (PBT) for BDIHCC.

**Methods:**

Between 2009 and 2018, 15 patients with BDIHCC underwent PBT at our institution. The overall survival (OS), local control (LC), and progression-free survival (PFS) curves were constructed using the Kaplan-Meier method. Toxicities were assessed using the Common Terminology Criteria of Adverse Events version 4.0.

**Results:**

The median follow-up time was 23.4 months (range, 7.9–54.3). The median age was 71 years (range, 58–90 years). Many patients were Child A (n = 8, 53.3%) and most had solitary tumors (n = 11, 73.3%). Additionally, most patients had central type BDI (n = 11, 73%). The median tumor size was 4.0 cm (range, 1.5–8.0 cm). The 1-, 2-, and 3-year OS rates were 80.0%, 58.7% and 40.2%, respectively, and the corresponding LC and PFS rates were 93.3%, 93.3%, and 74.7% and 72.7%, 9.7%, and 0.0%, respectively. Acute grade 1/2 dermatitis (n = 7, 46.7%), and grades 2 (n = 1, 6.7%) and 3 (n = 1, 6.7%) cholangitis were observed. Late toxicities such as grade 3 gastric hemorrhage and pleural effusion were observed. No toxicities of grade 4 or higher were observed.

**Conclusions:**

PBT was feasible with tolerable toxicities for the treatment of BDIHCC.

## Introduction

Hepatocellular carcinoma (HCC) with bile duct invasion (BDI) (BDIHCC) is rare, with a reported incidence of 0.5–13% according to the surgical findings [1] Surgical resection is generally considered the preferred treatment modality for patients with BDIHCC and those who undergo curative liver resection have been reported to gain a survival benefit [2, 3]. However, the prognoses of HCC patients with BDI have been reported to be worse than those of patients without BDI [2, 4, 5]. In addition, current guidelines do not provide recommendations regarding the specific treatment modality for BDIHCC. Moreover, application of non-surgical treatments for BDIHCC, such as radiofrequency ablation (RFA) and transcatheter arterial chemoembolization (TACE), is limited in some cases due to concerns about severe treatment-related biliary complications [6, 7].

Proton beam therapy (PBT) is a highly conformal radiotherapy that can deliver a high amount of energy over a target with a low exit dose [8, 9]. This remarkable feature of PBT makes it possible to treat HCC patients with varying tumor characteristics including location or size [10, 11]. Moreover, high LC with tolerable side effects were reported with the administration of PBT for the treatment of HCC [12–15]. Several studies also reported the outstanding outcomes of PBT in HCC patients with poor prognosis such as those with tumor thrombosis in portal vein and inferior vena cava [16, 17]. The current study aimed to evaluate the not-yet-explored safety and clinical outcomes of PBT in patients with BDIHCC.

## Methods

BDIHCC was evaluated through computed tomography (CT), magnetic resonance imaging (MRI), cholangiography, and magnetic resonance cholangiopancreatography. Cases with at least one of the following criteria were included in this study: [1] tumor thrombus within the bile duct or [2] tumor with infiltrative features associated with upstream biliary ductal dilatation. These images were independently analyzed by two radiologists. The inter-reader discrepancy was solved by subsequent mutual discussion and agreement. Based on the BDI classification system by Meng et al., we further classified BDIHCC into two types: central and peripheral BDI [4]. The central type BDI was defined as BDI of the common hepatic duct or first-order branch of the bile duct, and the peripheral type BDI was defined as BDI of second-order or higher peripheral branches of the bile duct without the invasion of the first-order branch or common hepatic bile duct.

### Study population

Between May 2009 and March 2018, 24 patients with BDIHCC underwent PBT at our institution. Nine patients were excluded from analysis: five due to extrahepatic metastasis, two due to incomplete data, and two due to treatment discontinuation. Finally, 15 patients were analyzed in this study. All these patients had localized HCC and were unfit for standared therapies because of their comorbidity, worse hepatic reserve, elderly age, concern for severe adverse events or patient’s reluctance to standard treatments. All study procedures involving human participants were conducted according to the ethical standards of the institutional research committee and the principles of the Declaration of Helsinki or its equivalent. This retrospective study was approved by the institutional review board of Tsukuba university hospital (R01-167). Written informed consent was originally obtained from every patient before the start of treatment, but such consent was waived for the current study.

### PBT

Fluoroscopically detectable metallic fiducial markers [1] were placed into the liver parenchyma beside the HCC under the ultrasound guidance. Surgical clips, stents and lipiodol accumulation were substituted for fiducial markers in patients who previously received treatment for HCC. A CT simulation scan was performed for patients who were immobilized in the supine position with respiratory synchronization during the expiratory phase at 2.5 or 5.0 mm intervals according to a previously established protocol [18] and treatment planning images were transferred to the VQA treatment planning system version 1.7 or 2.0 (Hitachi, Tokyo, Japan). The clinical target volume included visible tumors with a 5.0–10.0 mm margin. An aperture margin of 5.5–14.0 mm and an additional 0.0–5.0 mm margin in the direction of the caudal axis were added to encompass the entire clinical target volume.

### Follow-up of recurrence pattern and toxicity evaluation

Every two to four months after the end of PBT, laboratory data including the markers of liver function, serum alpha-fetoprotein and des-gamma-carboxy prothrombin were measured for all patients and abdominal CT or MRI was performed. Intrahepatic recurrences (IHR) were classified into early recurrence (< 24 months from the start of PBT) caused by micrometastases following treatment and late recurrence (≥ 24 monthss from the start of PBT) attributed to *de novo* tumors [19]. Local recurrence was defined as a tumor with continued growth in the irradiated field observed on CT or MRI [20]. Toxicity was assessed using the Common Terminology Criteria for Adverse Events version 4.0.

### Statistical analysis

Overall survival (OS), local control (LC), and progression-free survival (PFS) rates were estimated using the Kaplan-Meier method and the log-rank test was used to compare differences among survival curves. All time intervals were calculated from the first day of PBT. All statistical analyses were performed using R software version 4.0.5 (R Foundation for Statistical Computing, Vienna, Austria, https://R-project.org). *P*-values < 0.05 were considered statistically significant.

## Results

### Baseline patient characteristics

The median follow-up time was 23.4 months (range, 7.9–54.3). The median age was 71 years (range, 58–90), many patients were classified as Child class A (n = 8, 53.3%) and most had solitary tumors (n = 11, 73.3%). Moreover, most patients had central type BDI (n = 11, 73%) and 13 patients (86.7%) were diagnosed with HCC with bile duct tumor thrombosis (BDTT). A plastic stent was placed in three patients and a metallic stent in one patient due to bile duct obstruction and/or cholangitis before the start of PBT. The median tumor size was 4.0 cm (range, 1.5–8.0). Table 1 shows the patients’ baseline characteristics.

**Table 1:** The basic characteristics of the patients (n = 15)

**Basic characteristics**	
Age (years), median [IQR]	71 [63.5, 79.0]
Sex (Male / Female)	12 (80.0%) / 3 (20.0%)
Performance status score (0 / 1–3)	6 (40.0%) / 9 (60.0%)
Child (A / B,C)	8 (53.3%) / 7 (46.7%)
Etiology (HBV / HCV / NBNC)	2 (13.4%) / 8 (53.3%) / 5 (33.3%)
**Tumor characteristics**	
Size, median [IQR], cm	4.0 [3.4–6.3]
Number (Solitary / Multiple)	11 (73.3%) / 4 (26.7%)
Prior treatment history (No / Yes)	7 (46.7%) / 8 (53.3%)
Type of BDI (Central / Peripheral)	11 (73.3%) / 4 (26.7%)
BDTT (No / Yes)	2 (13.3%) / 13 (86.7%)
AFP, median [IQR], ng/ml	57.0 [15.7, 350.2]
DCP, median [IQR], mAU/ml	45 [17.0, 612.0]

Abbreviations: HBV, hepatitis B virus; HCV, hepatitis C virus; NBNC, non-HBV/non-HCV; BDI, bile duct invasion; BDTT, bile duct tumor thrombosis; AFP, alpha-fetoprotein; DCP, des-gamma-carboxy prothrombin

### Survival, LC, PFS, and recurrence pattern

**Fig. 1:**
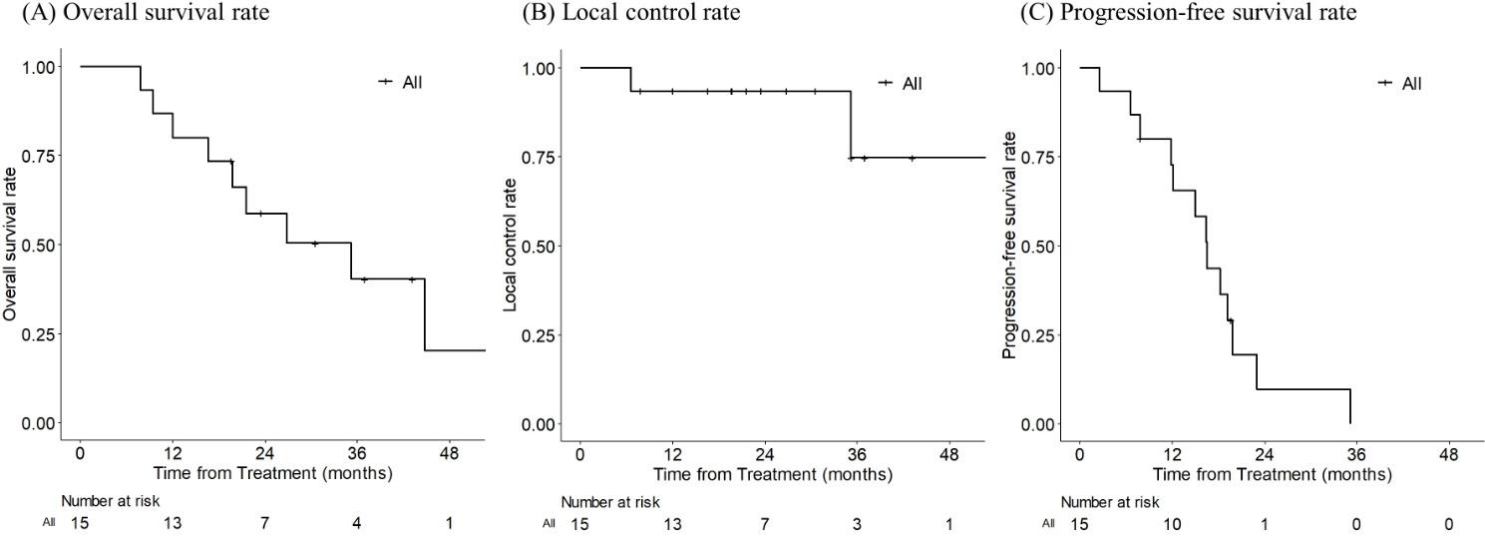
Clinical outcomes of patients receiving proton beam therapy for hepatocellular carcinoma with bile duct invasion. **(A)** Overall survival rates, **(B)** local control rates, and **(C)** progression-free survival rates

The 1-, 2-, and 3-year OS rates were 80.0%, 58.7% and 40.2%, respectively and the corresponding LC and PFS rates were 93.3%, 93.3%, and 74.7% and 72.7%, 9.7% and 0.0%, respectively (Fig. 1A-C). IHR alone was observed in seven patients, extrahepatic recurrence (EHR) alone was observed in one patient, both IHR and EHR were observed in three patients, and both IHR and local recurrence were observed in two patients. No recurrence was observed in two patients: a patient with Child-Pugh C liver cirrhosis was died of the progression of cirhossis within a year and another case was lost of follow-up after 19 months. Finally, a total of 12 (80.0%) patients developed IHR after PBT. Recurrence pattern, time from the start of PBT to first recurrence, and type of IHR were shown in Table 2.

**Table 2:** Recurrence pattern and classificaton of a type of recurrence interval

Age	Sex	Recurrence pattern	Time to the first recurrence (months)	Type of IHR
71	M	IHR and EHR	2.6	Early
80	M	IHR and LR	6.6	Early
63	M	IHR	7.8	Early
69	F	IHR	11.8	Early
63	M	IHR	12.1	Early
90	M	IHR	15.0	Early
73	M	IHR and EHR	16.4	Early
64	M	IHR and EHR	16.5	Early
58	M	EHR	18.2	NA
69	M	IHR and EHR	19.1	Early
78	M	IHR	19.9	Early
78	M	IHR	23.0	Early
86	F	IHR and LR	35.1	Late

Abbreviations: M, male; F, female; EHR. extrahepatic recurrence; IHR, intrahepatic recurrence; LR, local recurrence; NA, not applicable

**Fig. 2:**
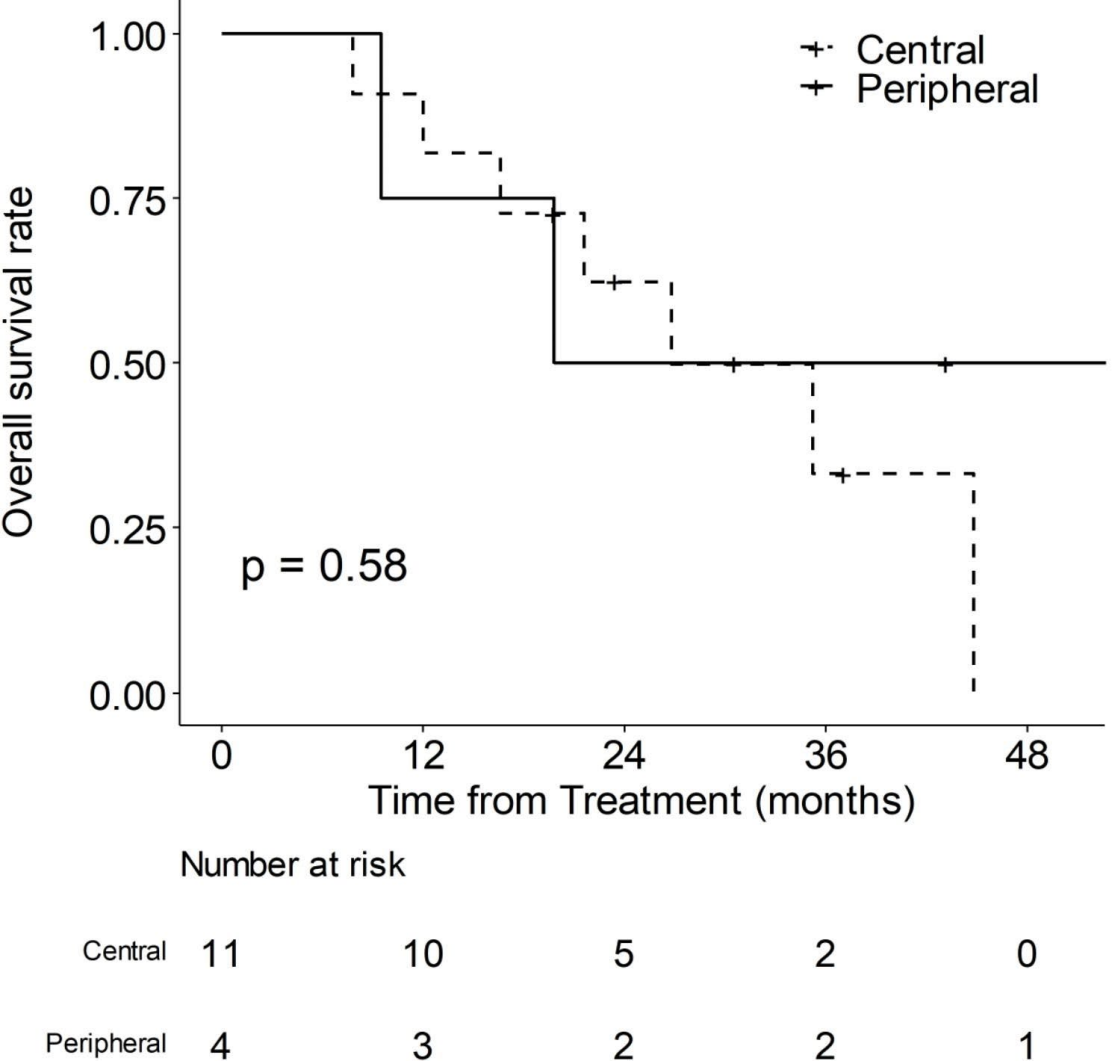
Overall survival rates according to types of hepatocellular carcinoma with bile duct invasion. Central type: dotted line, peripheral type: straight line

Table 3 shows basic characteristics of patients with central or peripheral BDIHCC. The 1-, 2-, and 3-year OS rates of central BDI were 81.8%, 62.3% and 33.2%, respectively and the corresponding rates of peripheral BDI were 75.0%, 50.0% and 50.0%, respectively. As shown in Fig. 2, significant differences in OS were not observed between the two types of BDI (*P* = 0.58).

**Table 3:** The basic characteristics of patients with hepatocellular carcinoma with central or peripheral invasion

Basic characteristics	Central (n = 11)	Peripheral (n = 4)	P value
Age (years), median [IQR]	69.0 [63.5, 80.0]	75.5 [70.5, 78.5]	0.69
Sex (Male / Female)	8 (72.7%) / 3 (27.3%)	4 (100.0%) / 0 (0.0%)	0.66
Performance status score (0 / 1–3)	4 (36.4%) / 6 (46.6%)	2 (50.0%) / 2 (50.0%)	0.36
Child (A / B,C)	5 (45.5%) / 6 (54.5%)	3 (75.0%) / 1 (25.0%)	0.56
Etiology (HBV / HCV / NBNC)	2 (18.2%) / 6 (54.5%) / 3 (27.3%)	3 (75.0%) / 1 (25.0%) / 0 (0.0%)	0.55
**Tumor characteristics**			
Size, median [IQR], cm	4.0 [3.5, 6.3]	4.4 [3.2, 6.0]	0.94
Number (Solitary / Multiple)	7 (63.6%) / 4 (36.4%)	4 (100.0%) / 0 (0.0%)	0.45
Prior treatment history (No / Yes)	5 (45.5%) / 6 (54.5%)	2 (50.0%) / 2 (50.0%)	0.73
BDTT (No / Yes)	0 (0.0%) / 11 (100.0%)	2 (50.0%) / 2 (50.0%)	0.10
AFP, median [IQR], ng/ml	57.0 [21.6, 315.5]	179.7 [6.3, 3130.7]	1.00
DCP, median [IQR], mAU/ml	48.0 [20.0, 612.0]	27.0 [17.5, 11731.8]	0.84

Abbreviations: HBV, hepatitis B virus; HCV, hepatitis C virus; NBNC, non-HBV/non-HCV; BDI, bile duct invasion; BDTT, bile duct tumor thrombosis; AFP, alpha-fetoprotein; DCP, des-gamma-carboxy prothrombin

### Toxicity

Acute grade 1/2 dermatitis (n = 7, 46.7%) and grades 2 (n = 1, 6.7%) and 3 (n = 1, 6.7%) cholangitis were observed. Late toxicities such as grade 3 gastric hemorrhage and pleural effusion were observed, while grade 4 or higher toxicity was not observed. Moreover, grade 3 or higher biliary stricture caused by PBT was not observed during the follow-up period. Finally, three patients developed hepatic failure due to disease progression. Acute and late toxicities are summarized in Table 4.

**Table 4:** Toxicities experienced by the patients (n = 15)

Acute	
Grade 1	Dermatitis (6)
Grade 2	Cholangitis (1), Dermatitis (1)
Grade 3	Cholangitis (1)
Late	
Grade 1	Hyperpigmentation (1), Telangiectasia (1), Radiation pneumonitis (1)
Grade 3	Gastric hemorrhage (1), Pleural effusion (1)

### Case presentation

**Fig. 3:**
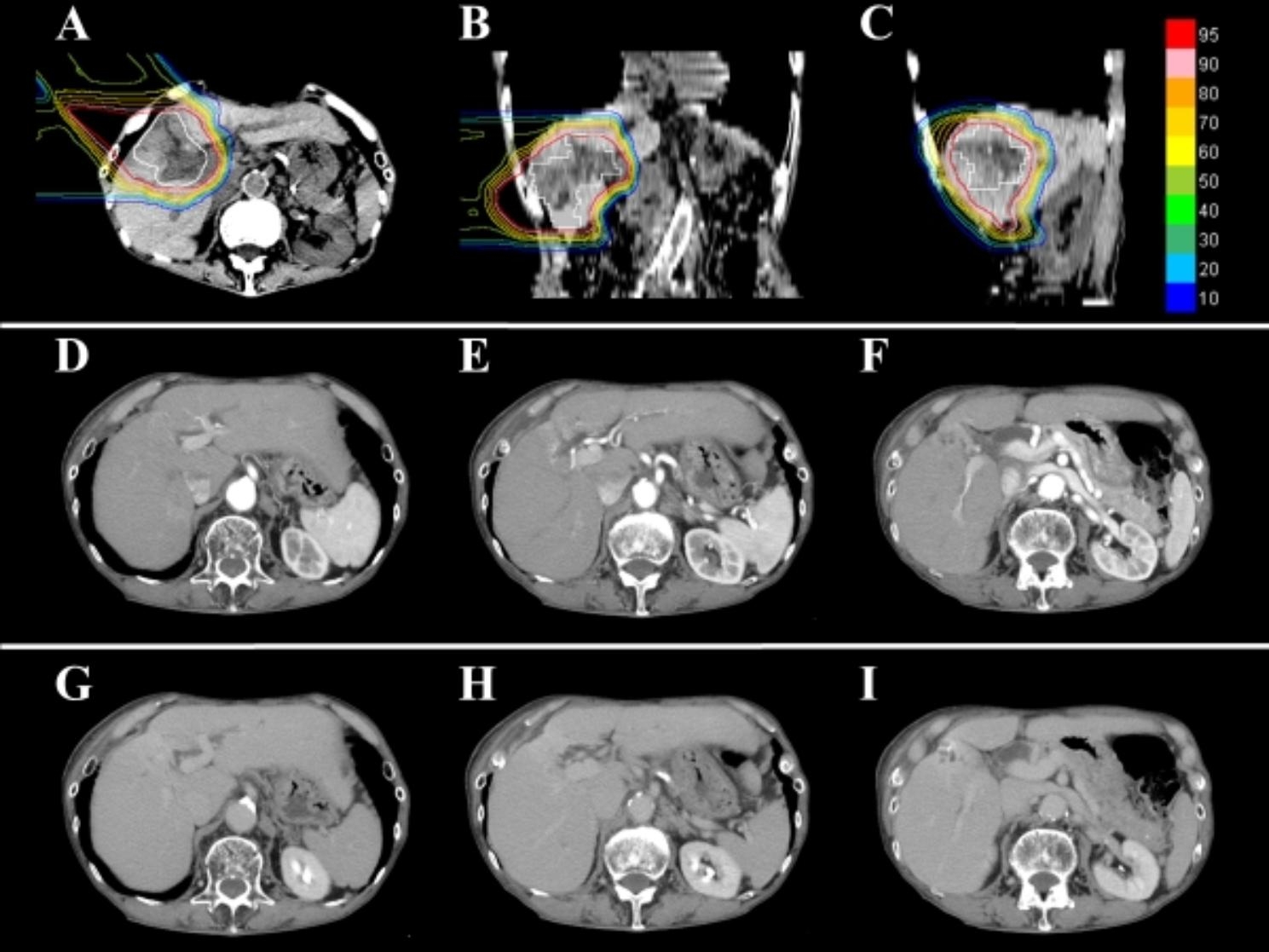
Pre-treatment computed tomography images of Case 1. **(A-F)** The arrowhead indicates bile duct tumor thrombus in axial slices. **(G)** The arrowhead indicates bile duct tumor thrombus in portal phase in a coronal slice. **(H)** The arrowhead indicates the location of bile duct tumor thrombus in magnetic resonance cholangiopancreatography

**Fig. 4:**
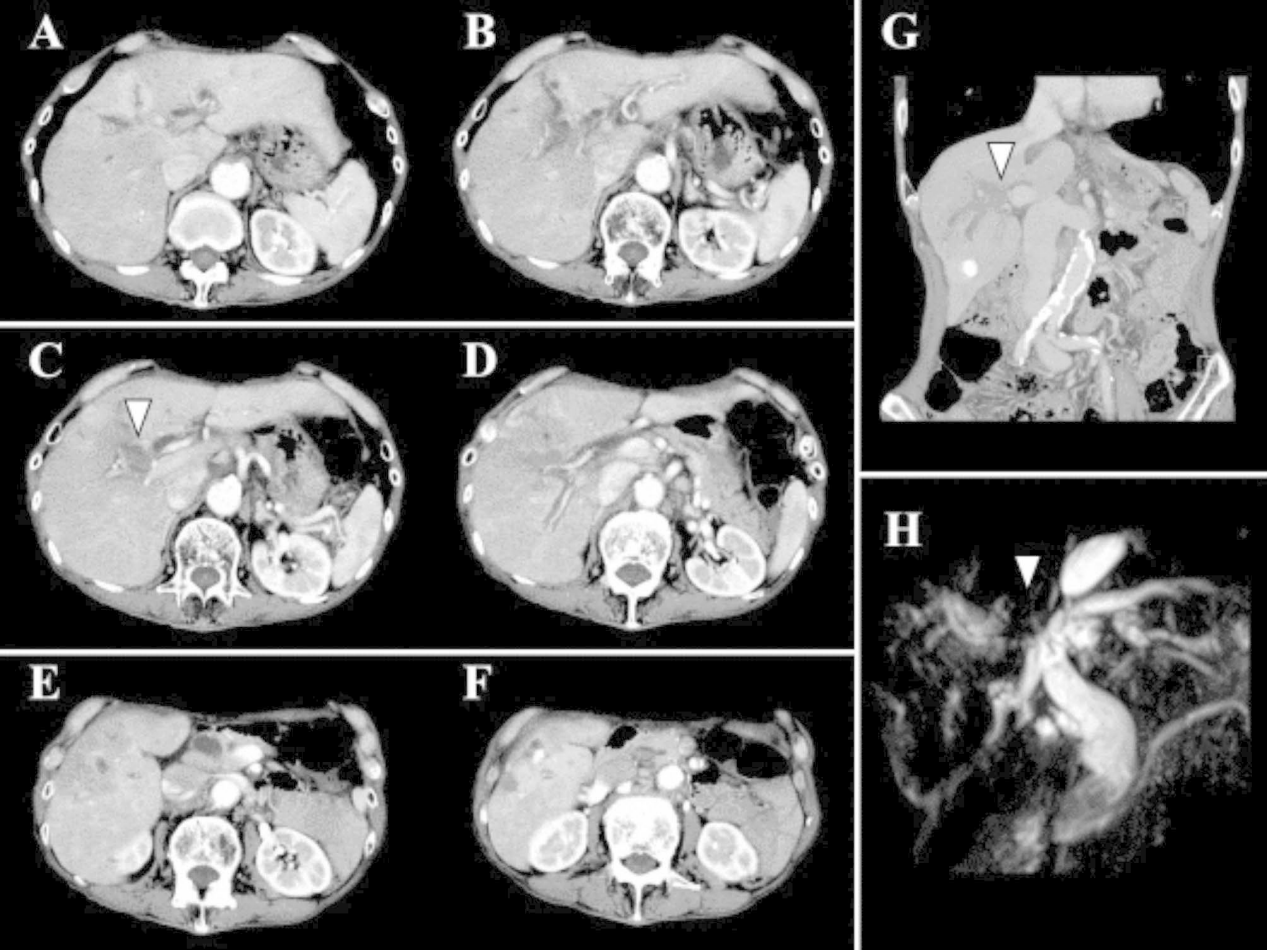
Proton beam therapy for Case 1 and computed tomography images during the follow-up period. **(A-C)** Dose distribution. The iso-dose lines represent 10–95% of the prescription dose from outside to inside. **(D-I)** Computed tomography images taken during follow-up 20 months from the start of proton beam therapy did not show any viable lesions and revealed the improvement of dilated bile duct in the liver parenchyma in early phase (D-E) and equilibrium phase (G-I).

A 90-year-old man with recurrent HCC in segment five had received two previous regimens of RFA a year and a half prior to presentation at our institution. The recurrent tumor was originated from the ablated area and the maximum tumor size was 5.5 cm in diameter. Mild enhancement during early phase and wash-out during portal and delayed phase were confirmed by contrast-enhanced CT. Additionally, BDTT in the right hepatic duct and dilatation of its distal bile ducts were observed using contrast-enhanced CT (Fig. 3A-G) and magnetic resonance cholangiopancreatography revealed a low signal filling defect of the same duct (Fig. 3H). The patient underwent PBT and a total dose of 72.6 Gy relative biological effectiveness was delivered in 22 fractions to the target (Fig. 4A-C). Fifteen months after the initiation of PBT, intrahepatic recurrence in segment two was observed and treated with TACE. The patient experienced complete remission, and dilatation of the bile ducts in the liver parenchyma was improved according to the follow-up CT taken at 20 months after PBT (Fig. 4D-I). Fifty-four months after the start of PBT, IHR in segment four was observed. Additionally, grade 1 acute dermatitis and other late adverse events, such as telangiectasia and hyperpigmentation were observed. The patient opted to only receive best supportive care due to his age. Finally, he died due to cardiovascular disease seventy-one months after the start of PBT.

## Discussion

Due to the paucity of reports on clinical outcomes of BDIHCC, a consensus has not yet been reached with regard to the prognostic impacts of BDI on patients with HCC or the management of BDIHCC. In the present study, we investigated survival rates, tumor control and adverse events of BDIHCC patients treated with PBT. Hence, the current study was the first to show the clinical outcomes and safety of PBT in patients with BDIHCC.

Surgical resection has been considered as the preferred treatment for BDIHCC. However, decreased patient survival and high recurrence rate after resection have been reported for patients with BDIHCC as shown in Table 5. The reported 1-, 2-, and 3-year OS rates were 21.9–69.2%, 11.0–53.8%, and 20.1–46.2%, respectively [2, 5, 21, 22]. As a result, the robust advantage of surgical resection for BDIHCC has not been established.

**Table 5:** Comparison of overall survival rates for the treatment of hepatocellular carcinoma with bile duct invasion

Author	Year	N	BDTT	Treatment	OS
Ikenaga, et al [1]	2009	15	NR	Surgery	1-yr 46%, 3-yr 23%, 5-yr 0%
Zeng, et al. [17]	2015	37	100%	Surgery	1-yr 64.2%, 2-yr 38.9%, 3-yr 24.3%
Jang, et al [18]	2015	13	NR	Surgery	1-yr 69.2%, 2-yr 53.8%, 3-yr 46.2%
Yang, et al [5]	2018	107	NR	Surgery	1-yr 60.5%, 3-yr 20.1%, 5-yr 12.0%
Current Study		15	86.7%	PBT	1-yr 80.0%, 2-yr 58.7%, 3-yr 40.2%

Abberviations: NR, not reported; PBT, proton beam therapy; OS, overall survival rates

In the current study, the 1-, 2-, and 3-year OS rates of PBT were 80.0%, 58.7%, and 40.2%, respectively. And these results were potentially comparable with the OS rates of surgical resection. The 1-, 2- and 3-year PFS rates were 72.7%, 9.7%, and 0.0%, respectively, which were worse than those of the previous report of PBT for patients with HCC from our intsitution [14]. Therefore, patients with BDIHCC have a high recurrence rate despite receiving curative PBT. Several studies have reported that BDI has a high risk for early IHR [2, 5, 23]. In the current study, IHR was observed in 12 patients (80.0%), of which 11 were classified as early recurrence attributed to micrometastases [19]. In addition, Ikenaga et al. also indicated that the infiltrative nature of BDIHCC was potentially caused by the tumor migration of HCC via the bile duct, leading to the worse prognosis of BDIHCC [2]. Moreover, a meta-analysis of 11 studies reported that HCC patients with BDTT had a worse prognosis than those without BDTT after surgical treatment (hazard ratio = 3.21, 95% confidence interval 2.34–4.39) [2]. In the present study, 83.7% patients were diagnosed with BDTT. Ikenaga et al. indicated that BDI upgraded tumor stage by one degree according to Japanese TNM classification and another study reported that the Japanese staging system reflected prognosis of patients more accurately than the American Joint Committee on Cancer/International Union Against Cancer staging systems that do not consider BDI for tumor staging [28].

As an effective way to achieve better clinical outcomes for early recurrence of BDIHCC, frequent follow-up and repeated surgery have been suggested in several studies [21, 24, 25]. Zeng et al. reported that recurrence after surgical resection of HCC associated with BDTT was a negative prognostic factor for OS and recurrence-free survival, and repeated surgical resections could prolong survival of such patients [21]. Peng et al. also reported that lower resectability and worse liver functions associated with underlying liver disease contributed to poor prognosis of BDTT [25]. Given that the repeated treatments contributed to the improvement of the prognosis of HCC with BDTT, PBT is a major potential option for BDIHCC because it allows to obtain a good LC and preserve liver function even after repeated PBT [26, 27]. Moreover, the combination usage of targeted therapies or immunotherapies with PBT for BDIHCC has a potentially crucial role in achieving better clinical efficacy. The high rates of LC with PBT for HCC is supposed to be compatible with systemic therapy by the prevention or improvement of obstructive jaundice. Therefore, further studies is required to fully verify the role of the combination therapy.

Ikenaga et al. reported that peripheral type BDI had an unfavorable impact on the prognosis of patients with HCC similar to that of central type BDI [2]. In the current study, a significant difference in OS was not observed between central and peripheral type BDI patients (p = 0.58). In accordance with these results, both type of BDI possibly lead to poor survival outcomes in patients with HCC underwent PBT.

Non-surgical treatment modalities such as RFA and TACE have a great risk of biliary complications in patients with BDIHCC. Furthermore, BDIHCC is a high risk factor of RFA-related complications. Puncture procedure and thermal damage during ablation can cause inadvertent intrahepatic bile duct injury [29]. Moreover, ablation of tumors in close proximity to the dilated bile duct is a predisposing factor for biliary tract infections [30, 31]. Additionally, TACE results in tumor control by the ischemic necrosis caused by embolization of the tumor-feeding hepatic arterial branch. However, the biliary ducts also have exclusive vascular supply from the branches of the hepatic artery [32]. Therefore, as a consequence of hepatic artery block, there is a high risk of unintentional occlusion of biliary blood supply, which can cause intrahepatic biloma and biliary stricture [7, 33]. These complications predispose patients to cholangitis and its sequelae, such as sepsis and hepatic failure, which can be fatal to patients with HCC [34]. While we observed two cases of manageable acute toxicities (grade 2 and 3 cholangitis), no grade 3 or higher late adverse events, such as biliary stricture were observed.

Park et al. reported clinical outcomes of chemoembolization for BDIHCC [1]. Three stratified groups according to biliary drainage and its effects were analyzed, and median OS periods were found to be 3.4–4.8 months. In our study, the median OS was 35.2 months. As a result, PBT appears to be an appropriate treatment modality for BDIHCC given its acceptable survival rates and tolerable adverse events.

The current study had several limitations including its retrospective nature and single-institutional data. Moreover, due to the rarity of BDIHCC this study involved a small sample, resulting in unavoidable selection bias. Based on our results, further multi-institutional studies are warranted to validate the outcomes of the current study. Furthermore, the evaluation of BDI and BDTT were based on imaging without pathological confirmation. However, in real clinical practice the diagnosis of HCC is made mostly based on imaging. Moreover, a retrospective study on the imaging features of BDTT suggested that intrahepatic dilation of the bile duct in HCC patients was useful in the diagnosis of BDTT with 93.33% sensitivity and 98.57% specificity [35]. Therefore, imaging modalities such as CT and MRI are considered to be appropriate for evaluating BDI and BDTT because they reflect clinical practice.

## Conclusions

PBT achieved relatively favorable survival rates without severe toxicities or biliary adverse events in BDIHCC patients. Therefore, radical PBT is a viable treatment for patients with BDIHCC.

## Data Availability

The data that support the finding of the current study are available from the corresponding author, TI, uppon reasonable request.
